# Dental Society Meetings

**Published:** 1882-07

**Authors:** 


"DENTAL SOCIETY MEETINGS," by our collaborator W. C. Barrett, M.D., which appears in this issue, we hope will be perused by all of our dental and medical readers. It is in *season* (as will be seen from the announcements which we made in June issue that quite a number of the dental societies meet in August), and points out the proper mode of discussing matters in medical and dental societies. On such occasions there is frequently too much airing "of knowledge in words of 'learned length and thundering sound.'" This is not only true in "elementary societies"—we have seen old heads in *august* meetings sit quiet and look wise under this hail of silvery knowledge.

Dr. Barrett says in his letter accompanying the article: "It is matter that is intended for the younger part of the profession and to such it may possibly be of interest. If it prove so I can work out the idea into another and perhaps more than one more article after the period for most of the States and United States meetings is over." To these promised papers our dental readers may look forward with pleasant anticipations.

Pardon our modesty (?) when we add Dr. Barrett's P.S.,—viz.: "THE INDEPENDENT PRACTITIONER ought to be supported by the profession for it is the exponent of a higher practice than is the most of our journals."

